# Early-onset adverse events after stereotactic radiosurgery for jugular foramen schwannoma: a mid-term follow-up single-center review of 46 cases

**DOI:** 10.1186/s13014-022-02057-8

**Published:** 2022-05-07

**Authors:** Young Goo Kim, Chang Kyu Park, Na Young Jung, Hyun Ho Jung, Jong Hee Chang, Jin Woo Chang, Won Seok Chang

**Affiliations:** 1 Department of Neurosurgery, Ewha Womans University Mokdong Hospital, Ewha Womans University College of Medicine, Seoul, Republic of Korea; 2 Kangwon National University College of Medicine, Chuncheon, Republic of Korea; 3 Department of Neurosurgery, Brain Research Institute, Yonsei Medical Gamma Knife Center, Yonsei University College of Medicine, 50 Yonsei-ro, Seodaemun-gu, Seoul, 03722 Republic of Korea; 4 Department of Neurosurgery, Kyung Hee University College of Medicine, Seoul, Republic of Korea; 5 Department of Neurosurgery, Ulsan University Hospital, Ulsan University College of Medicine, Ulsan, Republic of Korea

**Keywords:** Adverse effects, Stereotactic radiosurgery, Jugular foramen schwannoma, Radiation

## Abstract

**Background:**

Recently, stereotacitc radiosurgery (SRS) has been in the spotlight as an alternative therapeutic option for jugular foramen schwannomas (JFS). While most reported studies focus on the long-term efficacy and safety issues of SRS, none describe the early-onset adverse events (eAEs). We aimed to investigate the incidence, clinical characteristics, and mid-term outcomes of eAEs occurring within six months after SRS for JFS.

**Methods:**

In this retrospective review, patients who underwent at least six months of follow-up were included among all patients with JFS who have performed SRS at our institution between July 2008 and November 2019. And eAEs were defined as a newly developed neurological deficit or aggravation of pre-existing symptoms during the first six months after SRS.

**Results:**

Forty-six patients were included in the analysis. The median follow-up period was 50 months (range 9–136). The overall tumor control rate was 91.3%, and the actuarial 3-, 5-, and 10-year progression-free survival rates were 97.8%, 93.8%, and 76.9%, respectively. Of the 46 patients, 16 had eAEs, and the median time to onset of eAEs was one month (range 1–6 months), and the predominant symptoms were lower cranial nerve dysfunctions. Thirteen of 16 patients showed improved eAE symptoms during the follow-up period, and the median resolution time was six months (range 1–52). In 11 (68.8%) of 16 patients with eAEs, transient expansions were observed with a mean of 3.6 months after the onset of eAEs, and the mean difference between the initial tumor volume and the transient expansion volume was more prominent in the patients with eAEs (3.2 cm^3^ vs. 1.0 cm^3^; *p* = 0.057). In univariate analysis, dumbbell-shaped tumors (OR 10.56; *p* = 0.004) and initial tumor volume (OR 1.32; *p* = 0.033) were significantly associated with the occurrence of eAEs.

**Conclusions:**

Although acute adverse events after SRS for JFS are not rare, these acute effects were not permanent and mostly improved with the steroid treatment. Dumbell-shaped and large-volume tumors are significant predictive factors for the occurrence of eAEs. And the transient expansion also seems to be closely related to eAEs. Therefore, clinicians need to be more cautious when treating these patients and closely monitor the occurrence of eAEs.

## Background

Jugular foramen schwannoma (JFS) is a rare skull base tumor that accounts for 2.9-4.0% of all intracranial schwannomas [1]. JFSs are usually detected by radiological evaluation after patients gradually develop lower cranial nerve (CN) deficits. Because JFSs are benign, slow-growing tumors, complete surgical resection is considered an ideal curative treatment. However, despite recent advances in skull base microsurgical and intraoperative neuromonitoring techniques, total resection of JFSs without any neurological complications is challenging, even for highly experienced neurosurgeons, because of the anatomical location, which can be difficult to access surgically and the proximity of neurovascular structures [2–7].

During the last three decades, as with other brain tumors, such as vestibular schwannomas, steretactic radiosurgery (SRS) has been highlighted as an alternative treatment modality to primary microsurgery for small- to medium-sized JFSs or adjuvant therapy for residual or recurrent lesions because of the high tumor control rate with minimal morbidity [8]. However, while most previous studies have focused on the long-term efficacy and safety of SRS for JFSs, none have described early-onset adverse events (eAEs) that may cause a decline in patient compliance and deterioration of quality of life. Thus, this study aimed to investigate the incidence, clinical characteristics, and mid-term outcomes of eAEs occurring within six months after SRS for JFSs.

## Methods

### Patient population

This study was conducted on 46 patients with JFS who underwent SRS between July 2008 and November 2019 at Yonsei Gamma Knife Center. 31 of these patients underwent SRS as the primary treatment based on clinical presentations, and the following imaging criteria: (1) identified to be confined within the course of CN IX, X, or XI with intracranial extension with or without extracranial extension through the jugular foramen on thin-slice, axial T1-weighted images and constructive interference on steady-state images (or fast imaging employing steady-state acquisition); (2) did not have a salt-and-pepper appearance that is typical of glomus jugulare tumors [9], or the dural tail sign, which indicates meningioma; and (3) enlarged jugular foramen without bone destruction by the tumor. The remaining 15 patients had undergone prior microsurgery, and pathologic confirmation was schwannoma in all patients. Patients with neurofibromatosis type 2, and who underwent fractionated radiotherapy or staged SRS were excluded.

Tumors were defined as cystic when ≥ 25% of the tumor mass had a prominent cystic component. Eight tumors were cystic and 38 solid. Tumor locations were classified into four types, according to Kaye’s modified classification: primary intracranial (Type A), jugular foramen with intracranial extension (Type B), primary extracranial with foraminal extension (Type C), and intra-/extracranial extension (Type D) [1, 10].

### Radiosurgical techniques and follow-up evaluations

All SRS procedures were performed using the Leksell Gamma Knife Perfexion (Elekta AB, Stockholm, Sweden). All patients underwent magnetic resonance imaging (MRI) for target definition after applying the Leksell Model G stereotactic frame under local anesthesia. The treatment was planned based on MRI, considering the tumor size, vascularity, or bony destruction, using GammaPlan software (Elekta AB, Stockholm, Sweden). Tumor delineation and volume measurements were determined based on thin-slice, axial T1-weighted images with gadolinium enhancement and were modified using axial T2-weighted images. Before discharge following SRS, all patients were instructed to visit the hospital at any time after discharge, if pre-existing symptoms worsened or a new CN deficit occurred. All patients were followed up with clinical and neurological examinations one month after SRS, and then followed up every 6 or 12 months, depending on the patients’ condition. All patients underwent the first follow-up MRI within 6 months after SRS, and further follow-up MRI was routinely performed every 1 or 2 years, subsequently according to the patients’ condition. Tumour volumes were consistently measured by a single neurosurgeon (Y.G.K).

At our institute, we usually prescribe 13.0 Gy at the 50% isodose line; however, we adjusted the marginal dose (median 13.0 Gy; range, 12.0–14.0 Gy) according to the location and size of the JFS. The detailed demographic characteristics and radiosurgical treatment parameters are summarised in Table 1.

**Table 1:** Basic demographic characteristics and radiosurgical treatment parameters in 46 patients with jugular foramen schwannomas treated using gamma knife radiosurgery

Characteristics	Value
Median age at GKRS, years	49.5 (11–83)
Sex	
Male	18 (39.1)
Female	28 (60.9)
Laterality	
Left	28 (60.9)
Right	18 (39.1)
Pre-existing symptoms	27 (58.7)
CN VII–VIII^a^	3 (11.1)
CN IX–XII ^b^	16 (59.3)
CN VII–VIII and CN IX–XII	5 (18.5)
Neither CN VII–VIII nor CN IX–XII ^c^	3 (11.1)
Brainstem contact	29 (63.0)
Prior microsurgery	15 (32.6)
Tumour type^d^	
A: Primary intracranial	16 (34.8)
B: Jugular foramen with intracranial extension	3 (6.5)
C: Primary extracranial with foraminal extension	9 (19.6)
D: Intra/extracranial extension (dumb-bell shaped)	18 (39.1)
Median tumour volume, cm^3^	3.9 (0.2–13.7)
Median follow-up period, months	50 (9–136)
Median marginal dose, Gy	13.0 (12.0–14.0)
Median maximal dose, Gy	26.1 (24.0–28.7)

Values are expressed as numbers (%) or medians (range)

*GKRS* gamma knife radiosurgery

^a^Hoarseness, difficulty swallowing, tongue deviation, dysphagia, etc

^b^Hearing impairment, facial weakness, etc

^c^Headache, dizziness, etc

^d^Tumour types in this study according to Kaye’s modified classification

An eAE was defined as a newly developed neurological deficit or aggravation of pre-existing symptoms occurring within 6 months after SRS for JFS. In this study, most clinical information, such as the onset and disappearance of eAEs, was collected mainly based on the medical records and subjective patient self-reporting during the follow-up period. Using the final follow-up images, including contrast-enhanced thin-slice, axial T1-weighted images, complete remission was defined as tumor disappearance, partial remission was defined as a volume reduction of ≥ 25%, no change was defined as a volume reduction or increase of < 25%, and tumor progression was defined as a volume increase of ≥ 25% [11]. In this study, patients with complete remission, partial remission, or no change were considered the tumor control group. The transient expansion was deemed to be increased at least 10% tumor volume, followed by shrinkage during follow-up.

### Statistical analysis

Statistical analyses were performed using SPSS (version 23) (IBM Corp., Armonk, NY, USA). Descriptive statistics of categorical variables are presented as frequencies and percentages, and continuous variables are presented as medians and ranges. The Kolmogorov–Smirnov test was performed to test the normal distribution of the variables. Intergroup comparisons were performed using the Mann–Whitney *U* test and Student’s *t*-test for continuous variables, and Fisher’s exact test was used to compare categorical variables. After SRS, progression-free survival (PFS) was calculated using the Kaplan–Meier method. Univariate analysis was performed to assess the possible risk factors for eAEs after SRS. The strength of the association was measured using odds ratios (ORs) and their associated *p-*values. We did not perform multivariate analysis because of the insufficient sample size. Statistical significance was set at *p* < 0.05.

## Results

### Evaluation of pre-existing clinical symptoms

Twenty-seven (58.7%) of the 46 patients had pre-existing clinical symptoms at SRS. Among the symptoms, the most common were CN IX–XII-related symptoms (hoarseness, difficulty swallowing, tongue deviation, dysphagia, etc.) (n = 16). The remaining symptoms comprised CN VII–VIII-related symptoms (hearing impairment, facial weakness, etc.) (n = 3), CN VII–VIII- and CN IX–XII-related symptoms (n = 5), and neither CN VII–VIII nor CN IX–XII-related symptoms (headache, dizziness, etc.) (n = 3) (Table 1).

As indicated in Table 2, among the basic demographic characteristics and radiosurgical treatment parameters, in the pre-existing symptoms group, the median age at SRS and initial tumor volume were significantly higher than the non pre-existing symptoms group. A history of prior microsurgery was also more frequent in patients with pre-existing symptoms. Primary intracranial and dumbbell-shaped tumors were more frequent in the non pre-existing symptoms group and pre-existing group. At the last follow-up, among 27 patients with pre-existing clinical symptoms, 13 had improved, 12 were stable, and the remaining two had deteriorated.

**Table 2:** Basic demographic characteristics and radiosurgical treatment parameters according to the occurrence of pre-existing symptoms

Characteristics	Pre-existing symptoms group	Non pre-existing symptoms group	*P* value
Patients	27 (58.7)	19 (41.3)	
Median age at GKRS (year)	48 (13–83)	55 (11–76)	0.040^a^
Sex			0.767^b^
Male	10 (37.0)	8 (42.1)	
Female	17 (63.0)	11 (57.9)	
Laterality			0.767^b^
Left	17 (63.0)	11 (57.9)	
Right	10 (37.0)	8 (42.1)	
Tumour component			0.440^b^
Solid	21 (77.8)	17 (89.5)	
Cystic	6 (22.2)	2 (10.5)	
Brainstem contact	17 (63.0)	12 (63.2)	1.000^b^
Prior microsurgery	13 (48.1)	2 (10.6)	0.010^b^
Tumor type^c^			
A: Primary intracranial	5 (18.6)	11 (57.9)	0.006^a^
B: Jugular foramen with intracranial extension	2 (7.4)	1 (5.3)	1.000^a^
C: Primary extracranial with foraminal extension	6 (22.1)	3 (15.8)	0.774^a^
D: Intra-/extracranial extension (dumb-bell shaped)	14 (51.9)	4 (21.1)	0.034^a^
Median tumour volume (cm^3^)	4.2 (0.5–13.7)	1.7 (0.2–10.8)	0.010^a^
Median follow-up period, month	48 (9–135)	61 (24–136)	0.359^a^
Median marginal dose (Gy)	13.0 (12.0–14.0)	13.0 (12.0–14.0)	0.861^a^
Median maximal dose (Gy)	26.0 (24.0–28.7)	26.1 (24.2–28.5)	0.910^a^

Values are expressed as numbers (%) or medians (range)

*GKRS* gamma knife radiosurgery

^a^Statistical testing was performed using the Mann–Whitney *U* test and Student’s *t* test

^b^Statistical testing was performed using the Chi-square test or Fisher’s exact test

^c^Tumor types in this study according to Kaye’s modified classification

### Tumour control at last follow-up

**Fig. 1:**
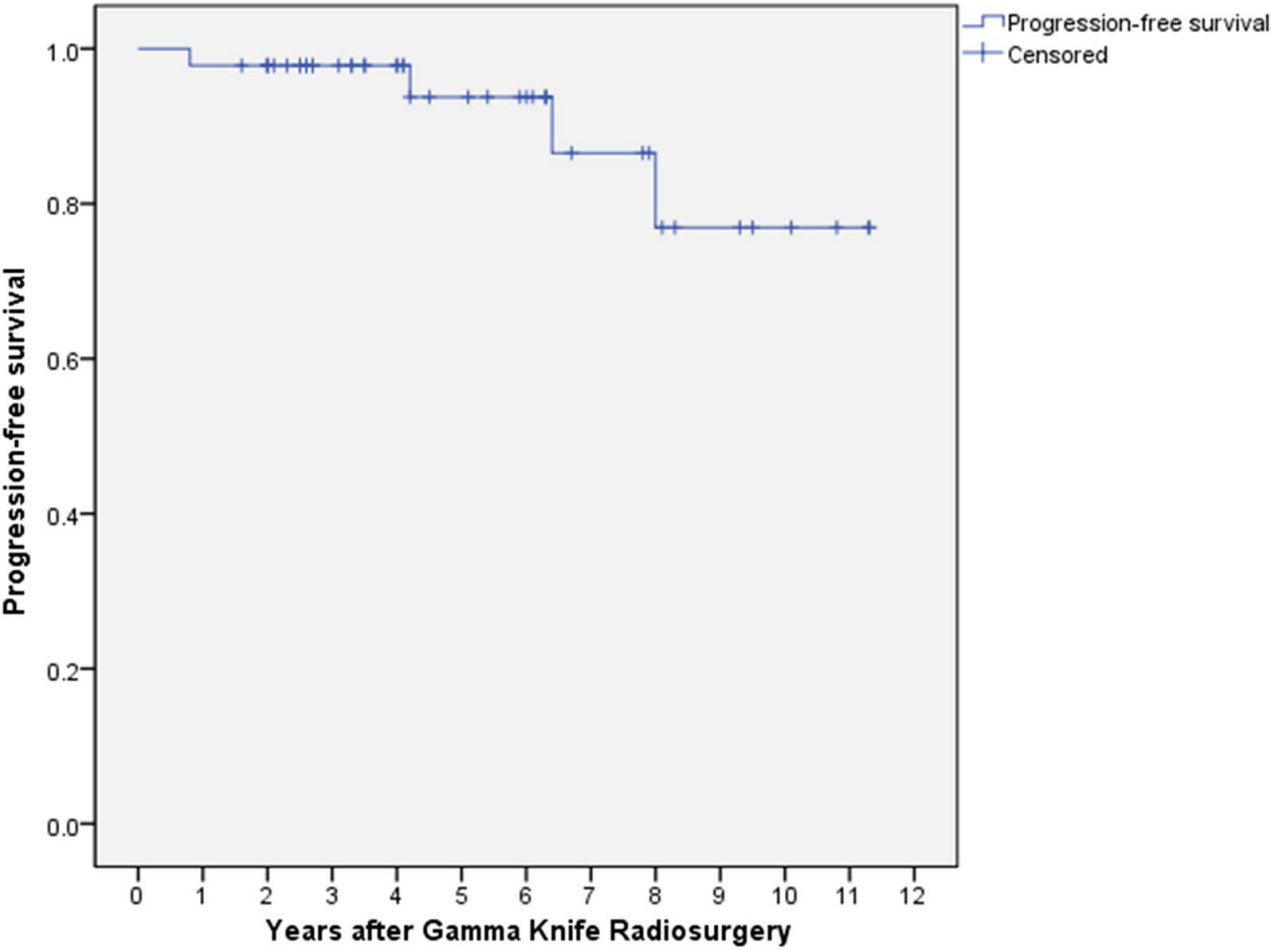
Kaplan–Meier curve for the actuarial tumor control rate in all patients with jugular foramen schwannoma after gamma knife radiosurgery

At the last follow-up, four (8.7%) of the 46 patients had tumor progression. 28 patients exhibited partial remission, and 14 patients showed no change in tumor size. Of the 4 patients with tumor progression, one patient died during follow-up due to a cause unrelated to JFS. The remaining patients were stable without any complications until the final follow-up. The actuarial 3-, 5-, and 10-year PFS rates were 97.8%, 93.8%, and 76.9%, respectively (Fig. 1). On follow-up imaging, transient expansion occurred in 16 of the 46 patients, with a median time to tumor enlargement of 6 months (range 3–14 months) after SRS; and the median time to the disappearance of transient expansion was 10 months (range 4–44 months).

### Clinical characteristics of patients with early-onset adverse events

Of the 46 patients, 16 had eAEs, and the median time to onset of eAEs was 1 month (range 1–6 months). Among the basic demographic characteristics and radiosurgical treatment parameters, dumbbell-shaped tumors (intra-/extracranial extension [Type D]) were significantly more frequent in the eAE group. The initial tumor volume was significantly higher in the eAE group than in the non-eAE group. Interestingly, the transient expansion was more pronounced in the eAE group. Of the 16 patients with eAEs, transient expansion was observed in 11 patients (Table 3). In these patients, the median time to the appearance and disappearance of transient expansion was 5.5 months (range 3–6 months) and 9 months (range 4–36 months) after SRS. Although not statistically significant, the mean difference between the initial tumor volume and the transient expansion volume was more prominent among patients with eAEs (3.2 cm^3^ vs. 1.0 cm^3^; *p* = 0.057). In the eAE group, new symptoms developed in nine patients, while aggravation of pre-existing symptoms occurred in seven patients. The most common symptoms in both groups were CN IX–XII-related symptoms, including hoarseness, difficulty swallowing, and tongue deviation (Table 4).

**Table 3:** Basic demographic characteristics and radiosurgical treatment parameters according to the occurrence of early-onset adverse radiation effects

Characteristics	eAEs group	Non-eAEs group	*P* value
Patients	16	30	
Median age at GKRS, years	49 (21–76)	48 (11–83)	0.533^a^
Sex (%)			0.210^b^
Male	4 (29.4)	14 (46.7)	
Female	12 (70.6)	16 (53.3)	
Laterality (%)			0.347^b^
Left	7 (43.8)	20 (66.7)	
Right	9 (56.2)	10 (33.3)	
Tumour component (%)			0.694^b^
Solid	14 (87.5)	24 (80.0)	
Cystic	2 (12.5)	6 (20.0)	
Brainstem contact (%)	11 (68.8)	18 (60.0)	0.750^b^
Prior microsurgery (%)	5 (31.3)	10 (33.3)	1.000^b^
Transient expansion (%)	11 (68.8)	5 (16.7)	0.001^b^
Tumour location ^c^ (%)			
A: Primary intracranial	2 (12.5)	14 (46.7)	0.026^b^
B: Jugular foramen with intracranial extension	1 (6.3)	2 (6.7)	1.000^b^
C: Primary extracranial with foraminal extension	2 (12.5)	7 (23.3)	0.463^b^
D: Intra-/extracranial extension (dumb-bell shaped)	11 (68.8)	7 (23.3)	0.004^b^
Median tumour volume, cm^3^	4.85 (2.4–10.8)	3.1 (0.2–12.3)	0.003^a^
Median follow-up period, months	48.5 (24–136)	52 (9–123)	0.729^a^
Median marginal dose, Gy	13.0 (12.0–14.0)	13.0 (12.0–14.0)	0.868^a^
Median maximal dose, Gy	26.2 (24.4–28.7)	26.1 (24.0–28.7)	0.641^a^

Values are expressed as numbers (%) or medians (range)

*eAEs* early-onset adverse events, *GKRS* gamma knife radiosurgery

^a^Statistical testing was performed using the Mann–Whitney *U* test and Student’s *t-*test

^b^Statistical testing was performed using the chi-square test or Fisher’s exact test

^c^Tumour types in this study according to Kaye’s modified classification

**Table 4:** Type of early-onset adverse events after gamma knife radiosurgery (classified as the dominant symptom)

Variable	Patients, n (%)
Newly developed symptoms	9 (56.3)
CN VII–VIII^a^	1 (6.2)
CN IX–XII^b^	5 (31.3)
CN VII–VIII and CN IX–XII	3 (18.8)
Neither CN VII–VIII nor CN IX–XII^c^	0 (0.0)
Aggravation of pre-existing symptoms	7 (43.7)
CN VII–VIII^a^	0 (0.0)
CN IX–XII^b^	5 (31.2)
CN VII–VIII and CN IX–XII	2 (12.5)
Neither CN VII–VIII nor CN IX–XII^c^	0 (0.0)

*CN* cranial nerve

^a^Hearing impairment, facial weakness, etc

^b^Hoarseness, difficulty swallowing, tongue deviation, dysphagia, etc

^c^Headache, dizziness, etc

When eAEs were clinically observed, intravenous or oral corticosteroid therapy was initiated and usually continued for approximately 1 or 2 weeks. Subsequently, these patients were symptomatically treated for each symptom (ex. vocal cord injection, temporary Levin tube insertion, antibiotics administration, etc.). Symptoms of eAEs were resolved in 13 of 16 patients, of whom seven were in the new-onset symptoms group, and six were in the exacerbation of pre-existing symptoms group. None of the patients developed aspiration pneumonia in this study, which required additional tracheostomy or gastrostomy. The median resolution time was 6 months (range 1–52 months). Although there was a difference in symptom severity, the remaining three patients, including two patients with deteriorated pre-existing symptoms and one with new-onset symptoms, complained that symptoms related to eAEs continued to the final follow-up, despite appropriate treatment. Of the 22 patients in the non-eAE group, two developed a CN deficit (at 11 and 12 months after SRS, respectively); none of their symptoms had improved at the last follow-up. In univariate analysis, dumbbell-shaped tumors (OR 10.56; *p* = 0.004) and initial tumor volume (OR 1.32; *p* = 0.033) were significantly associated with the occurrence of eAEs. (Table 5).

**Table 5:** Results of the univariate analysis for predictive factors of early-onset adverse events

Variable	OR	95% CI	*P *value
Age	1.01	0.970–1.043	0.755
Sex (male)	0.40	0.098–1.636	0.202
Laterality (Rt)	3.43	0.878–13.390	0.076
Tumour component (solid)	0.68	0.119–3.933	0.671
Brainstem contact	1.26	0.320–4.939	0.743
Prior microsurgery	1.21	0.295–4.982	0.790
Dumbbell-shaped tumor ^a^	10.56	2.167–51.420	0.004
Initial tumour volume (cm^3^)	1.32	1.022–1.694	0.033
Marginal dose (Gy)	1.22	0.344–4.302	0.761
Maximal dose (Gy)	1.36	0.747–2.492	0.312

*OR* odds ratio, *CI* confidence interval

^a^ Tumor types in this study according to Kaye’s modified classification

## Discussion

### Clinical presentation

Although schwannoma is the third most common benign central nervous system tumor after meningioma and pituitary adenoma, schwannomas arising from the glossopharyngeal, vagus, or accessory CNs are rare, constituting only 2.9–4.0% of all intracranial schwannomas [12, 13]. The most common clinical symptoms in patients with JFSs are lower CN dysfunction, such as difficulty swallowing, hoarseness, or tongue deviation after additional tumor growth [7, 12, 14]. Similarly, among the 27 patients with pre-existing clinical symptoms in this study, the most common was a deficit of CN IX–XII (16 patients; 59.3%), followed by CN VII–VIII and CN IX–XII (5 patients; 18.5%). Patients with pre-existing symptoms were younger, had larger initial tumor volumes, and had more experience with prior microsurgeries (Table 2). This means that a large initial tumor volume can cause early detected symptoms associated with JFS, leading to earlier surgical treatment.

### Stereotactic radiosurgery

Schwannomas are typically benign nerve sheath tumors composed of Schwann cells, which produce the insulating myelin sheath covering peripheral nerves, and are relatively slow-growing and typically contained within a capsule; thus, surgical resection is often successful [15]. However, even for highly experienced neurosurgeons, it is challenging to completely remove schwannomas arising from CNs IX, X, and XI, without any complications, despite the recent advances in skull base surgical techniques and neuromonitoring because of their rarity, anatomical location, and relationship to adjacent critical structures. In an earlier reported case series, Pluchino et al. [16] reported a mortality rate of 16%. In a literature review, the same authors also documented a mortality rate of 9%. Although lower mortality rates have been reported in recently published microsurgical case series, serious complications such as CN deficits are still described. In recent studies [1, 3, 6, 7, 17, 18], transient CN VII and VIII deficits after surgical resection varied from 11 to 80% and 8–45%, respectively, and permanent deficits were reported in 4–20% and 4–20%, respectively. Transient lower CN deficits varied from 25 to 60%, and permanent deficits varied from 10 to 48%. With recent advances in neuroimaging modalities, such as computed tomography and MRI, the number of incidentally detected tumors in asymptomatic patients is likely to increase. For asymptomatic patients, postoperative complications such as CN VII–VIII and lower CN injury, cerebrospinal fluid leakage, and infection, could be an even more serious issue.

Recently, SRS has been highlighted as a minimally invasive alternative to microsurgery for JFSs, or adjuvant treatment, because of the high tumor control rate and low incidence of severe complications [8, 11, 19–21]. In a 2007 study, Martin et al. [19] reported 34 patients (35 tumors) with JFS who underwent SRS. The 5- and 10-year actuarial PFS rates were 97% and 94%, respectively, with a mean follow-up of 84 months. Worsening of pre-existing lower CN deficits occurred in a single patient whose increased tumor volume led to early surgical resection. Recently, Hasegawa et al. [14] and Kano et al. [22] published an 18-institution Japanese multicentre report and an international multicentre report, respectively. In a Japanese study [14], the authors reported their experience with 117 patients with JFS who underwent SRS with a median follow-up of 52 months (range 12–248 months). Partial remission and stable tumors were observed in 62, and 42 patients, respectively, and tumor progression was detected in 13 patients. The 3- and 5-year PFS rates were 91% and 89%, respectively. During the follow-up period, eight patients developed persistent symptomatic deterioration after SRS. The cause was tumor progression in four patients and adverse radiation effects in four patients. In a nine-institution international multicentre JFS study, Kano et al. [22] reported tumor regression in 47/92 patients, stable tumors in 33/92 patients, and progression in 12/92 patients, with a median follow-up of 51 months (range 6–266 months). The 3-, 5-, and 10-year PFS rates were 93%, 87%, and 82%, respectively. Fourteen patients had delayed onset of additional CN symptoms. In this study, 42 of the 46 patients exhibited partial remission or no change in tumor size, and four patients had tumor progression at the last follow-up. The 3-, 5-, 10-year PFS rates were 97.8%, 93.8%, and 76.9%, respectively, with a median follow-up of 50 months (range 9–136 months). At the last follow-up, five patients reported persistent symptomatic deterioration, including two with delayed onset (> 6 months) adverse events.

### Early-onset adverse events after stereotactic radiosurgery

The most significant difference between our study and the previously published studies is that our study focused on the onset and resolution of eAEs that occurred during the first 6 months after SRS. Of the 46 patients with JFS who underwent GKRS, 16 developed eAEs (median onset time, 1 month; range 1–6 months). Symptoms of eAE were resolved in 13 of these 16 patients (median resolution time, 6 months; range 1–52 months) by the last follow-up.

In this study, regarding the characteristics of adverse events, the evaluation of eAEs (occurrence, improvement, and type of symptom) was mainly based on the patient’s subjective experience of discomfort. Because radiation concentrated on the JFS mainly affects the lower CNs, consequently, radiation-related functional deterioration may be more diverse in patients with JFS than in those with vestibular schwannoma. And, it is also difficult to immediately and objectively evaluate lower CN dysfunction, such as difficulty swallowing and dysphagia, in patients who are followed up after SRS at outpatient clinics, unlike facial palsy or hearing loss. In a study by Hasegawa et al. [14], clinical follow-up data were obtained from referring doctors, and when clinically indicated, Kano et al. [22] assessed CN and other neurological functions using additional measures, such as facial electromyography, audiograms, and dynamic swallowing tests. Although quantitative assessment of radiation-related adverse events may have been possible in these studies, however, the actual discomfort experienced by the patients is likely underestimated. Therefore, it is also important to carefully assess subjective discomfort.

In this study, univariate analysis showed that dumbbell-shaped tumors and initial tumor volume were significantly associated with eAEs (Table 5). Kano et al. [22] reported that dumbbell-shaped tumors were significantly larger than non-dumbbell-shaped tumors (7.7 vs. 3.3 cm^3^; *p* < 0.001) and were significantly associated with a higher rate of symptomatic deterioration because non-dumbbell-shaped tumors are recognized at an earlier stage, which facilitates earlier and more successful management with SRS. Similar results were observed in the present study. The median initial volume of dumbbell-shaped tumors was also considerably larger than that of non-dumbbell-shaped tumors (5.1 vs. 2.8 cm^3^; *p* = 0.008), and the median maximal dose for dumbbell-shaped tumors was higher (26.4 vs. 26.0 Gy; *p* = 0.003) (Table 6). These findings suggest that dumbbell-shaped tumors were significantly associated with a higher rate of eAEs, because their larger volumes were managed with a higher maximal dose.

**Table 6:** Radiosurgical treatment parameters according to tumor location

Characteristics	Dumbbell shaped tumor	Non-dumbbell shaped tumor	*P* value
	n = 18	n = 28	
Median age at GKRS, years	49 (21–74)	51 (11–83)	0.210^a^
Median tumour volume, cm^3^ (range)	5.1 (2.2–13.7)	2.8 (0.2–9.9)	0.008^a^
Median follow-up period, months (range)	45 (9–136)	52 (19–135)	0.324^a^
Median marginal dose, Gy (range)	13.0 (12.0–14.0)	13.0 (12.0–14.0)	0.149^a^
Median maximal dose, Gy (range)	26.4 (25.2–28.7)	26.0 (24.0–28.7)	0.001^a^

Values are expressed as medians (range)

*GKRS* gamma knife radiosurgery

^a^Statistical testing was performed using the Mann–Whitney *U* test and Student’s *t-test*

Martin et al. [19] reported that JFSs undergo temporary volume enlargement between 6 and 18 months after radiosurgery, and surgical resection is seldom necessary because this effect tends to decline over additional follow-up. A transient increase in the size of schwannomas followed by stability or regression has been increasingly recognized following stereotactic radiosurgery for vestibular schwannoma, termed pseudo-progression or tumor swelling [23]. Pseudo-progression or tumor swelling is not usually considered a treatment failure because, as mentioned above, the tumor tends to shrink during follow-up [4, 23–27]. In this study, transient volume expansion was also observed in 16 of 46 patients, and of the 16 patients with eAE, transient expansion occurred in 11. The transient expansion was observed with a mean of 3.6 months after the onset of eAEs. Compared to the initial tumor volume in patients with transient expansion, the mean increased tumor volume was greater in patients with eAEs. These facts suggest that transient expansion may be a possible factor affecting the development of eAEs.

### Study limitations

This study has several limitations. First, this study was inherently limited in its retrospective design. Second, the relatively small sample size may limit the generalisability of the results. Third, this study analyzed results from a single center without multicentre involvement. Fourth, as mentioned above, in this study, the assessment of eAEs was based on subjective patient self-reporting without using measurement tools. Finally, 31 of the 46 patients were diagnosed using radiological findings and clinical history only; therefore, these may not be cases of JFS. Despite these limitations, our study has several advantages over previous studies in that the present study focused on acute clinical adverse events in the short-term period after SRS for JFS and the associated risk factors. This single-center experience could provide valuable information regarding the eAEs, leading to a decline in patient compliance and deterioration of quality of life. To our best knowledge, this study is the first of its kind.

## Conclusions

SRS provided reasonable tumor control in most patients with either primary or residual JFS. This study indicates although acute adverse events occurring within the first 6 months after SRS for JFS are not rare, these acute effects were not permanent and mostly improved with the steroid treatment. Dumbell-shaped and large-volume tumors are statistically significant factors for the occurrence of eAEs. Additionally, even though transient expansion is not a predictive factor of the eAEs, it could be considered closely related to eAEs. Clinicians need to be more cautious when treating these patients and should closely monitor the occurrence of eAEs during follow-up.

## Abbreviations

SRS: Stereotactic radiosurgery
JFS: Jugular foramen schwannoma
eAE: Early-onset adverse event
MRI: Magnetic resonance imaging
OR: Odd ratio
CN: Cranial nerve
PFS: Progression-free survival
